# Total Bilirubin Predicts Severe Progression of Diabetic Retinopathy and the Possible Causal Mechanism

**DOI:** 10.1155/2020/7219852

**Published:** 2020-07-31

**Authors:** Yu Ding, Junmin Zhao, Gangsheng Liu, Yinglong Li, Jiang Jiang, Yun Meng, Tingting Xu, Kaifeng Wu

**Affiliations:** Department of Ophthalmology, Hefei Binhu Hospital, The Third Affiliated Hospital of Anhui Medical University, Hefei, Anhui 230001, China

## Abstract

Early detection and treatment are key to delaying the progression of diabetic retinopathy (DR), avoiding loss of vision, and reducing the burden of advanced disease. Our study is aimed at determining if total bilirubin has a predictive value for DR progression and exploring the potential mechanism involved in this pathogenesis. A total of 540 patients with nonproliferative diabetic retinopathy (NPDR) were enrolled between July 2014 and September 2016 and assigned into a progression group (*N* = 67) or a stable group (*N* = 473) based on the occurrence of diabetic macular edema (DME), vitreous hemorrhage, retinal detachment, or other conditions that may cause severe loss of vision following a telephonic interview in August 2019. After further communication, 108 patients consented to an outpatient consultation between September and November 2019. Our findings suggest the following: (1) TBIL were significant independent predictors of DR progression (HR: 0.70, 95% CI: 0.54–0.89, *p* = 0.006). (2) Examination of outpatients indicated that compared to stable group patients, progression group patients had more components of urobilinogen and LPS but a lower concentration of TBIL. The relationship between bilirubin and severe DR was statistically significant after adjusting for sex, age, diabetes duration, type of diabetes, FPG, and HbA1c (OR: 0.70, 95% CI: 0.912–0.986, *p* = 0.016). The addition of serum LPS and/or urobilinogen attenuated this association. This study concludes that total bilirubin predicts an increased risk of severe DR progression. Decreasing bilirubin might be attributed to the increased levels of LPS and urobilinogen, which may indicate that the change of bilirubin levels is secondary to intestinal flora disorder and/or intestinal barrier destruction. Further prospective investigations are necessary to explore the causal associations for flora disorder, intestinal barrier destruction, and DR.

## 1. Introduction

Due to the aging of population, urbanization, and lifestyle changes, diabetes mellitus (DM) has increased rapidly in both developed and developing countries. Diabetic retinopathy (DR) is the most common microvascular complication of diabetes and is the main cause of blindness among working-age people in industrialized countries [1]. In the early stage of nonproliferative diabetic retinopathy (NPDR), chronic hyperglycemia can damage the microvessels supplying the retina, leading to ischemia, vascular leakage, and central vision loss caused by diabetic macular edema (DME) [2]. As the disease progresses to proliferative DR (PDR), vision loss associated with secondary neovascularization of the retina and subsequent hemorrhage and/or retinal detachment occurs [3]. Previous studies have shown that DR progression and vision loss can impair the patient's quality of life; however, without appropriate intervention, about half of high-risk PDR patients will develop visual impairment due to DME, vitreous hemorrhage, and/or retinal detachment within five years of diagnosis [4]. Therefore, early detection and treatment are vital for delaying the progression of DR, avoiding vision loss, and reducing the burden of advanced disease.

Bilirubin has effective antioxidant properties and is a protective agent against diabetes and cardiovascular diseases [5]. A meta-analysis assessing 132,240 subjects recruited from 27 studies found a negative nonlinear relationship between bilirubin concentration and the risk of diabetic complications [6]. Prospective studies showed that the level of serum total bilirubin was independently correlated with DR in both Type-1 Diabetes Mellitus (T1DM) and Type-2 Diabetes Mellitus (T2DM) [7]. However, there is no current consensus on lower serum total bilirubin having predictive value for the development of DR. Further, the biological mechanisms for the relationship between serum bilirubin levels and DR remain unclear.

Therefore, the present study examined whether total bilirubin has a predictive value for DR progression. The potential mechanism involved in this pathogenesis was also examined.

## 2. Materials and Methods

### 2.1. Participants

In this study, a total of 540 patients with an initial diagnosis of NPDR were enrolled in the Department of Endocrinology, the Third Affiliated Hospital of Anhui Medical University (Hefei Binhu Hospital) between July 2014 and September 2016. Retinopathy was diagnosed by binocular indirect slit-lamp fundoscopy and fundus photography after mydriasis with eye drops containing 0.5% tropicamide and 5% phenylephrine by a single grader. The final diagnosis of DR was made by fundus photographs. Color fundus photographs of two fields (macular field, disc/nasal field) of both eyes were taken with a 45 fundus camera (VISUCAM, Zeiss), according to the EURODIAB retinal photography methodology [3]. In this study, NPDR was defined as the presence of one or more microaneurysms, hemorrhages, and/or hard exudates. These evaluations were performed independently by two different ophthalmologists after training. After a telephone interview in August 2019, based on having DME, vitreous hemorrhage, retinal detachment, or other conditions induced by diabetes that caused severe vision loss, patients were assigned into a progression group (*N* = 67; among them, 47 patients were diagnosed in our hospital, and the rest were diagnosed in other hospitals at the same level) or a stable group (*N* = 473). The exclusion criteria were as follows: (1) no history of T1DM or T2DM; (2) vision loss due to nondiabetic causes during pregnancy or lactation or both; (3) presence of cancer, hepatic disease, or other coexisting illnesses, including a history of coronary stent implantation, cerebral infarction, and severe CKD (defined as eGFR ≤ 30 ml/min/1.73 m^2^); and (4) inability to communicate using standard methods. After further communication, 108 patients were scheduled to visit as outpatients between September and November in 2019. Written informed consent was obtained from subjects or parents/legal guardians during the outpatient visit.

### 2.2. Methods

**Figure 1:**
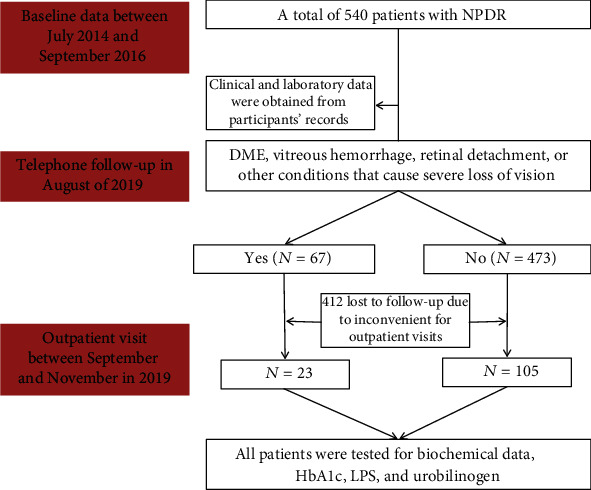
Flow chart of inclusion participants.

Data regarding the duration and type of diabetes, along with age and gender, were obtained from participants' medical records. All patients were tested for biochemical data (ALT, AST, Cr, TBILI, DBIL, IBIL, *γ*-GT, and ALP) and glucose metabolism (FPG, HbA1c) to obtain baseline levels. Outpatient visits were performed between September and November 2019. Overnight fasting blood samples (patients fasted for at least 8 hr.) were taken, and plasma from these samples was examined to obtain biochemical data such as HbA1c and lipopolysaccharide (LPS). The eGFR was calculated as follows: 194 × Cr^−1.094^ × age^−0.287^ (×0.739 for female patients). CKD stage was divided into six categories based on eGFR levels as follows: G1, ≥90; G2, 60–89; G3a, 45–59; G3b, 30–44; G4, 15–29; and G5, <15 ml/min/1.73 m^2^. Urine protein was divided into three categories as follows: normoalbuminuria (urinary albumin-to-creatinine ratio (UACR), <30 mg/gCr); microalbuminuria (UACR, 30–299 mg/gCr), and macroalbuminuria (UACR, ≥300 mg/gCr). First-morning urine samples were collected to test for urobilinogen. Urobilinogen in urine was measured by direct spectrophotometry using a modified Ehrlich's method. A LPS/LOS ELISA Kit (USCN Life Science Inc., Houston, Texas, USA) was used to estimate the concentration of plasma LPS. A flow chart of the process is shown in Figure 1.

### 2.3. Statistical Analysis

IBM SPSS Statistics ver. 22.0 (IBM Co., Armonk, NY, USA) was used. Continuous measurements, such as the mean (SD), were utilized if data were normally distributed; however, if the data were not normally distributed, the median (IQR) was utilized. Categorical variables were described utilizing frequency and percentages (%). Independent tests, including the *t*-test, chi-square test, or Mann-Whitney *U* test, were used to compare the two patient groups. A Cox proportional hazards regression model to estimate the hazard ratio (HR) with 95% CI was used to analyze the risk factors for DR progression, or stability. Kaplan-Meier survival curves of NPDR progression by serum TBIL stratification were determined. Logistic regression analysis was adopted to calculate the odds ratio (OR) and a 95% confidence interval (CI) for the risk of DR progression, and this was determined after adjusting for potential confounding variables. Statistical significance was set at *p* < 0.05.

## 3. Results

### 3.1. Demographic and Metabolism Characterization of Study Subjects

Data from a total of 540 patients with DR (47.41% male and 20.74% T1DM) were evaluated. The average follow-up time was 48 (40–54) months, and the mean age of the population was 61.36 ± 15.49 years. Subject age varied from 24 to 87 years. The patients had been diabetic for 0–37 years. Patients in the progression group had a significantly higher duration of diabetes and lower TBIL, DBIL, IBIL, and FPG than those in the stable group (both *p* < 0.05). There were no significant differences in sex, age, type of diabetes, HbA1c, ALT, AST, *γ*-GT, ALP, and Cr between the two groups (all *p* > 0.05) (Table 1).

### 3.2. Independent Risk Factors Associated with the Progression of DR

**Figure 2:**
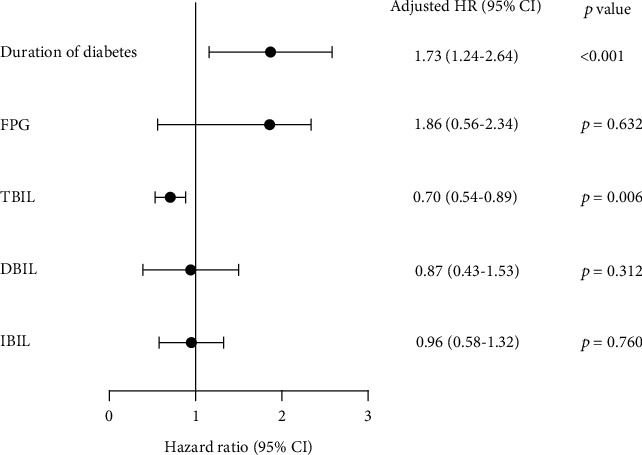
Cox proportional hazards regression model: the risk factors for DR progression.

The Cox proportional hazards regression model was used to analyze the risk factors for DR progression. After adjusting for all factors with significant associations emerging from the univariate analysis, duration of diabetes and TBIL were significant independent predictors of DR progression (HR: 1.73, 95% CI: 1.24–2.64, *p* < 0.001; HR: 0.70, 95% CI: 0.54–0.89, *p* = 0.006, respectively), as presented in Figure 2.

### 3.3. Kaplan-Meier Survival Curves of NPDR Progression by Serum TBIL Stratification

**Figure 3:**
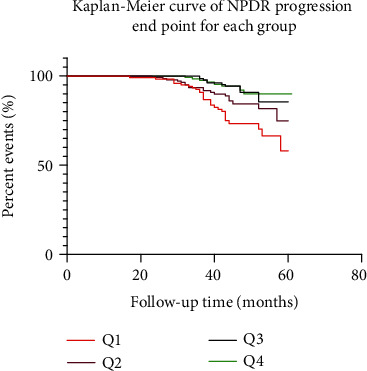
Kaplan-Meier survival curves by TBIL stratification (*n* = 540). Patients were categorized into four groups: Q1 (*N* = 121), Q2 (*N* = 140), Q3 (*N* = 151), and Q4 (*N* = 128).

As mentioned above, serum TBIL was correlated with NPDR progression outcome. A further analysis with TBIL stratification was performed to estimate the ratio in different serum TBIL levels. TBIL was categorized into four groups according to the interquartile range as follows: Q1 (*N* = 121), <9.45; Q2 (*N* = 140), 9.45–10.30; Q3 (*N* = 151), 10.30–11.20; and Q4 (*N* = 128), ≥11.20 *μ*mol/l. The Q2, Q3, and Q4 groups were significantly different when compared with the Q1 group (log-rank *χ*^2^ = 4.85, *p* = 0.0277; *χ*^2^ = 16.03, *p* < 0.001; and *χ*^2^ = 15.07, *p* < 0.001, respectively) (Figure 3).

### 3.4. Characterization of DR Individuals on Outpatient Visit

After further communication, 108 patients (23 from the progression group and 105 from the stable group) visited as outpatients between September and November 2019. The study groups were similar as regards age, gender distribution, diabetes type, and duration. The progression group had more components of urobilinogen and LPS but lower concentrations of TBIL. The general data and data on biochemical indexes of the two groups are summarized in Table 2.

### 3.5. Odds Ratios for Severe DR

To explore the effects of bilirubin on DR progression, we performed multivariable analyses using logistic regression models. The relationship between bilirubin and severe DR was statistically significant after adjusting for sex, age, diabetes duration, type of diabetes, FPG, and HbA1c (odds ratio (OR): 0.967, 95% CI: 0.912–0.986, *p* = 0.016; Model 1). The addition of serum LPS (Model 2), urobilinogen (Model 3), LPS, and urobilinogen (Model 4) in Model 1 attenuated this association. The details of the models are summarized in Table 3.

### 3.6. Relationship between TBIL, Urobilinogen, and LPS

Table 4 shows a strong negative correlation between TBIL/urobilinogen (*r* = −0.796, *p* < 0.001) and TBIL/LPS (*r* = −0.708, *p* < 0.001) in DR individuals.

## 4. Discussion

Previous studies have shown serum bilirubin plays a protective role against the development of diabetic microvascular complications, such as neuropathy, nephropathy, and DR [8]. To our knowledge, this is the first study that investigates the predictive value of serum total bilirubin level at risk of DR progression. In Cox proportional hazards regression analysis, the duration of diabetes and serum total bilirubin level were independently related to DR progression. Subsequent Kaplan-Meier survival analysis with stratification by TBIL interquartile range indicates that the Q2, Q3, and Q4 groups were significantly different when compared with the Q1 group in the incidence of DR progression. The outpatient visit shows that the relationship between bilirubin and severe DR was statistically significant after adjusting for sex, age, diabetes duration, type of diabetes, FPG, and HbA1c (OR = 0.967); however, the addition of serum LPS and/or urobilinogen to a model utilized in this work attenuated the association. A further analysis indicates a strong negative correlation between TBIL/urobilinogen (*r* = −0.796) and TBIL/LPS (*r* = −0.708) in those DR individuals.

In recent years, diabetes has become a rapidly growing threat around the world. Many of its complications not only cause a significant burden but also has an important impact on physical health and quality of life [9]. Therefore, it is important to identify diabetic individuals with a higher risk of complications. This may improve prevention and reduce the burden of the disease. DR is a common and special microvascular complication that develops with the passage of time [10]. Severe stages of DR, including DME and PDR, lead to visual impairment and blindness [11]. Epidemiological studies have shown that about one in three diabetic patients suffers from DR, and one in ten has PDR or DME. Demographic surveys indicate that half of the population suffering from diabetes have not been diagnosed. In addition, individuals with an early stage of DR have not been given sufficient attention [3]. In the current study, at approximately four years subsequent to the first diagnosis of NPDR, 12.41% of the 540 patients developed severe DR, such as DME, vitreous hemorrhage, retinal detachment, or other conditions that led to severe vision loss. Consequently, it is vital to find predictive factors for desire progression during the early course of DR.

A majority of previous studies suggested a negative relationship between TBIL and DR [8, 12]. A study of T2DM patients indicated that a higher TBIL was independently associated with a reduced risk of DR (OR: 0.242, 95% CI: 0.096-0.615) [13]. A population-based cross-sectional study indicated that patients with a serum bilirubin level in the fourth quartile were less likely to develop DR than those in the first quartile for serum bilirubin level (OR: 0.55; 95% CI: 0.33~0.91) [14]. A meta-analysis indicated a significant, nonlinear, and negative correlation between TBIL and DR risk (OR: 0.19, 95% CI: 0.14-0.25) [15]. However, there are few studies regarding the predictive value of bilirubin in the development of NPDR [16]. In a prospective cohort study, a higher baseline bilirubin level was associated with a significantly reduced risk of progression from microalbuminuria to macroalbuminuria [17]. Our results are consistent with this study, indicating that serum bilirubin concentration is negatively correlated with the development of NPDR and may be a useful predictor of serious progress of DR over time.

The pathogenesis of DR has not been adequately studied. Oxidative stress caused by high glucose is an area of focus in current studies [18]. TBIL is not only a metabolite of hemoglobin but also an important endogenous antioxidant [19]. Previous studies have demonstrated that TBIL has a significant protective effect against cardiovascular disease, diabetes, and diabetic macrovascular complication. This is a result of antioxidant and anti-inflammatory effects [20]. As a natural antioxidant, uric acid has similar antioxidant effects, such as resisting oxidative stress, scavenging oxygen-free radicals, preventing apoptosis, and protecting vascular endothelial cells from DNA [21]. A large number of studies have shown that higher levels of serum uric acid are associated with a higher risk of DR [22–24]. Those findings seem to contradict previous studies regarding the antioxidant effects of bilirubin on the pathogenesis of DR. This suggests that other potential mechanisms are probably involved in this process.

Anatomically, the portal system transports intestinal blood to the liver, which contains not only nutrients but also molecular patterns related to pathogens, including LPS, and peptidoglycan among other substances [25]. Studies in rodents and humans have shown that a long-term, high-fat diet (HFD) can lead to intestinal barrier defects, which may promote the transport of intestinal contents (food antigens, bacterial by-products, and bacteria themselves), especially bacterial LPS, into systemic circulation, resulting in low-grade inflammation [26]. Previous studies have also demonstrated that obese individuals and animals fed HFD exhibit changes in the composition of intestinal microflora and a two-three factor increase in serum LPS concentration [27]. In our study, the progressive group had more LPS components, suggesting that these patients may be experiencing more severe intestinal flora disorders and intestinal barrier disruptions. Urobilinogen refers to a group of colorless tetrapyrrole formed when intestinal anaerobes reduce intestinal unconjugated bilirubin (conjugated bilirubin secreted to the upper small intestine is hydrolyzed to unconjugated bilirubin). Up to 20% of the urine bilirubin produced daily is reabsorbed from the intestine and undergoes enterohepatic recirculation [28]. Most of the reabsorbed urobilinogen is taken up by the liver and subsequently reexcreted into bile, while a small amount is excreted into the urine. In this study, we found that the progression group had a higher level of urobilinogen but a lower concentration of TBIL when compared to the stable group. The relationship between bilirubin and DR progression was statistically significant after adjusting for known risk factors; however, the addition of serum LPS or urobilinogen in the corresponding model attenuated this association. These results may support the assertion that for intestinal flora disorder and intestinal barrier destruction, the reabsorption of urobilinogen will be increased through enterohepatic circulation or local damage of the vascular barrier which would lead to the positive feedback of bilirubin excretion. In other words, the decreasing of bilirubin is secondary to intestinal flora disorder and/or intestinal barrier destruction.

Several limitations of this study should be mentioned. First, as this was a single-centre study in China, the results might not be directly applicable to other ethnicities and regions. Second, there were 20 patients diagnosed with DR progression in other hospitals, which might have led to potential heterogeneity. Finally, unmeasured confounding factors might not have been fully addressed.

## 5. Conclusions

In conclusion, our study indicates a negative relationship between total bilirubin concentration and the progression of DR, which might be attributed to the increased levels of LPS and urobilinogen. This may indicate that a decrease of bilirubin is secondary to intestinal flora disorder and/or intestinal barrier destruction. Further prospective investigations are necessary to explore the causal associations for flora disorder, intestinal barrier destruction, and DR.

## Figures and Tables

**Table 1:** Demographic and metabolism characterization of study subjects.

Group	Progression group (*N* = 67)	Stable group (*N* = 473)	*T*/*F*/*χ*^2^ value	*p* value
General data				
Gender (male/female)	32/35	224/249	0.004	0.951
Age (year)	62.34 ± 19.78	61.12 ± 13.27	0.657	0.512
Average follow-up time (month)	49 (40–56)	47 (38–54)	0.301	0.743
Duration of diabetes (year)	12.79 ± 6.31	10.12 ± 4.29	4.46	<0.001
Type of diabetes (T1DM/T2DM)	19/48	93/380	2.79	0.248
Glucose metabolism				
FPG (mmol/l)	8.92 ± 1.97	8.43 ± 1.88	1.985	0.048
HbA1c (%)	8.69 ± 3.06	8.64 ± 3.77	0.104	0.917
Biochemical data				
ALT (IU/l)	18 (13–24)	16 (10–22)	0.258	0.784
AST (IU/l)	17 (9-26)	17 (8-30)	0.135	0.876
TBIL (*μ*mol/l)	8.36 ± 2.89	11.36 ± 3.65	6.450	<0.001
DBIL (*μ*mol/l)	1.38 ± 0.74	2.3 ± 1.04	6.992	<0.001
IBIL (*μ*mol/l)	6.98 ± 1.89	8.06 ± 3.57	2.427	0.016
*Γ*-GT (IU/l)	18 (14–27)	19 (15–30)	0.368	0.697
ALP (IU/l)	102 (72–140)	92 (83–147)	1.368	0.205
Cr (*μ*mol/l)	86 (32–102)	81 (37–98)	1.231	0.274
eGFR (ml/min/1.73 m^2^)	61.43 ± 13.12	62.56 ± 14.37	0.609	0.543
CKD stage (number)			0.333	0.954
G1	12	97		
G2	23	162		
G3a	18	125		
G3b	14	89		
Urine protein categories (number)			0.395	0.821
Normoalbuminuria	39	287		
Microalbuminuria	21	147		
Macroalbuminuria	7	39		

**Table 2:** Characterization of DR individuals in an outpatient visit.

Group	Progression group (*N* = 23)	Stable group (*N* = 105)	*T*/*F*/*χ*^2^ value	*p* value
Gender (male/female)	9/14	53/52	0.972	0.324
Age (year)	65.14 ± 19.43	67.09 ± 23.75	0.367	0.714
Average follow-up time (month)	50 (39–55)	49 (37–54)	0.269	0.835
Duration of diabetes (year)	13.76 ± 6.47	12.46 ± 5.93	0.937	0.351
Type of diabetes (T1DM/T2DM)	6/17	28/77	0.003	0.955
ALT (IU/l)	21 (13–39)	19 (14–33)	0.943	0.316
TBIL (*μ*mol/l)	8.04 ± 3.14	12.46 ± 3.42	6.147	<0.001
*Γ*-GT (IU/l)	21.5 (10–36)	19 (12–35)	1.845	0.124
Cr (*μ*mol/l)	91 (45–106)	87 (42–104)	1.654	0.189
eGFR	57.46 ± 16.35	59.04 ± 14.27	0.468	0.640
FPG (mmol/l)	8.14 ± 3.17	7.06 ± 2.38	1.850	0.067
HbA1c (%)	8.19 ± 2.76	7.93 ± 1.87	0.55	0.583
Urobilinogen (mg/dl)	0.75 (0.23–1.04)	0.48 (0.05–0.67)	5.568	<0.001
LPS (Eu/ml)	0.71 (0.34–1.79)	0.58 (0.20–1.45)	2.263	0.037

**Table 3:** Odds ratios for severe DR.

	Bilirubin (*μ*mol/l)	*p* value	LPS (Eu/ml)	*p* value	Urobilinogen (mg/dl)	*p* value
Unadjusted	0.894 (0.765–0.943)	<0.001	—	—	—	—
Model 1	0.967 (0.912–0.986)	0.016	—	—	—	—
Model 2	0.969 (0.934–1.023)	0.084	2.476 (1.632–3.091)	<0.001	—	—
Model 3	0.992 (0.960–1.104)	0.136	—	—	1.734 (1.234–2.430)	0.009
Model 4	1.013 (0.893–1.347)	0.422	1.985 (1.346–2.808)	0.016	1.702 (1.141–2.336)	0.027

Model 1: adjusted for sex, age, diabetes duration, type of diabetes, FPG, and Hba1c; Model 2: Model 1+LPS; Model 3: Model 1+urobilinogen; Model 4: Model 1+LPS+urobilinogen.

**Table 4:** Relationship between TBIL, urobilinogen, and LPS.

Variable	TBIL	
	*β*	*p* value
Urobilinogen	-0.796	0.000
LPS	-0.708	0.000

## Data Availability

The datasets analyzed during the current study are available from the corresponding author.
